# Optical Coherence Tomography versus Visual Evoked Potentials in detecting subclinical visual impairment in multiple sclerosis

**Published:** 2014

**Authors:** M Grecescu

**Affiliations:** * Ophthalmology Department, Bucharest Emergency University Hospital, Bucharest, Romania

**Keywords:** Optic coherence tomography (OCT), visual evoked potential (VEP), Retinal nerve fiber layer (RNFL), multiple sclerosis (MS)

## Abstract

**Rationale.** Visual impairment is one of the most common clinical manifestations of multiple sclerosis (MS). Some multiple sclerosis patients complain of poor vision although the Snellen visual acuity is 20/20. This study reveals that sensitive measurements like visual evoked potential (VEP) and optical coherence tomography (OCT) can evidence subclinical disturbances of visual pathway. These methods examine the relation between the visual function (VEP) and retinal nerve fiber layer (RNFL) thickness, as a structural biomarker for axonal loss in patients with multiple sclerosis (MS). The findings in this study indicate the utility of combining structural and functional testing in clinical research on patients with MS.

**Purpose.** To detect visual impairment in a population of visually asymptomatic patients affected by clinically definite multiple sclerosis (MS) and to compare the utility of optical coherence tomography (OCT) versus visual evoked potentials (VEP).

**Material and methods.** Fourteen patients (28 eyes) affected by clinically definite MS, without a history of optic neuritis and asymptomatic for visual disturbances, were initially fully examined (visual acuity, ocular fundus, biomicroscopy) from an ophthalmic point of view and then measured by OCT (RNFL thickness) and VEP.

Patients with a history of glaucoma or other retinal or optic nerve disease were excluded.

**Results.** Of fourteen patients (28 eyes), VEP was abnormal in 11 cases (78,57%) and OCT (RNFL thickness) was abnormal in 5 cases (35,71%), while 3 patients had no abnormalities on neither tests.

**Conclusions.** Optical coherence tomography (OCT) is less sensitive than visual evoked potentials (VEPs) in detecting visual subclinical impairment in patients with multiple sclerosis (MS). VEP remains the preferred test for the detection of clinical and subclinical optic neuritis. OCT may provide complementary information to VEP in cases with clinical definite MS and represent a valuable research instrument for the study of optic nerve disease in populations. The findings in this study reveal the utility of combining structural and functional testing in clinical research on patients with MS.

**Abbreviations**: multiple sclerosis = MS, optical coherence tomography = OCT, visual evoked potential = VEP, retinal nerve fiber layer = RNFL, best corrected visual acuity = BCVA.

## Introduction

Visual dysfunction is one of the most common clinical manifestations of multiple sclerosis (MS). Just over a decade ago, MS clinical trials did not include visual outcomes, but experts recognized the need for more sensitive measures of visual function [**3**].

Optical coherence tomography (OCT) is a noninvasive high-resolution technique that uses near-infrared light to generate cross-sectional tomographic images of tissues, including the retinal nerve fiber layer (RNFL) [**3**,**4**]. Optical coherence tomography is used to monitor retinal ganglion cell axon loss in glaucoma, diabetic retinopathy, traumatic optic neuropathy, chiasmal lesions and optic neuritis [**4**-**8**]. Recently, OCT has been studied in patients with multiple sclerosis (MS). Two studies showed that the eyes without a history of optic neuritis among MS patients have decreased RNFL thickness compared with the eyes of control subjects, suggesting that retinal ganglion cell axonal loss occurs separately from acute optic neuritis in MS patients [**3**,**11**,**13**].

This suggests that OCT can be used to monitor axonal injury and visual dysfunction in MS and may be an useful outcome measure in clinical trials [**6**-**11**,**14**].

The alteration in visual evoked potential (VEP) latencies by using pattern stimuli is considered one of the most characteristic electrophysiological signs observed in patients with MS [**15**]. In this study, we compared OCT versus VEP in the detection of visual dysfunctions in a population of visually asymptomatic patients with clinically definite MS.

## Material and methods

Fourteen patients (28 eyes) diagnosed with clinically definite multiple sclerosis and no visual impairment and optic neuritis history, were selected from the Department of Neurology of the Bucharest Emergency University Hospital. In all cases, the disease was in remission. The tests used to detect the involvement of visual pathway were optical coherence tomography (OCT) and visual evoked potential (VEP).

All the patients underwent full ophthalmic examination, including:

• Best corrected visual acuity (BCVA)

• Biomicroscopy

• Ocular fundus examination after pharmacologically pupil dilatation

The Snellen visual acuity equivalent was determined by the lowest line read on the 100% chart.

An important exclusion criteria of the patients after the ophthalmic examination was the presence of nystagmus, which can have an important effect on visual fixation (an essential component in obtaining high-quality OCT scans).

The retinal nerve fiber layer (RNFL) measurements were obtained after 3 consecutive scans centered on the optic nerve head. OCT software Cirrus, Carl Zeiss Meditec generated a mean RNFL thickness measurement for 360 degrees around the optic disc, four retinal quadrants and 12 clock for our segment (30 degrees for each hour position). All scans were performed without pupil dilatation [**4**].

VEPs were elicited by checkerboards reversing at 2 Hz (2 reversals per second) on a television monitor located 1 m from the patient.

VEP measurements were obtained with the patient wearing a visual aid, if needed and the fellow eye was occluded. A fixation spot was used on the center of the screen during stimulation. The latency of the first major positive peak in the VEP (P100 wave) was measured. Most investigators currently interpreted pattern reversal VEP based almost entirely on the latency of P100. A P100 latency >115ms was considered abnormal [**15**].

## Results

The best-corrected visual acuity (BCVA) was 20/20 (Snellen acuity) in all cases.

Biomicroscopy and ocular fundus examination after pupil dilatation showed no abnormalities.

This study revealed that 5 out of 14 patients had a RNFL mean reduction around 12%, assuming a normal RNFL thickness around 100 µm (97µm). The resulted fitting parameters were very similar:

• 1 patient had a RNFL loss of 5 µm (RNFL thickness = 92 µm)

• 1 patient had a RNFL loss of 7 µm (RNFL thickness = 90 µm)

• 1 patient had a RNFL loss of 8 µm (RNFL thickness = 87 µm)

• 1 patient had a RNFL loss of 13 µm (RNFL thickness = 84 µm)

• 1 patient had a RNFL loss of 15 µm (RNFL thickness = 82 µm)

The mean peripapillary RNFL thinning was of 87 µm for eyes without history of optic neuritis. 

**Fig. 1:**
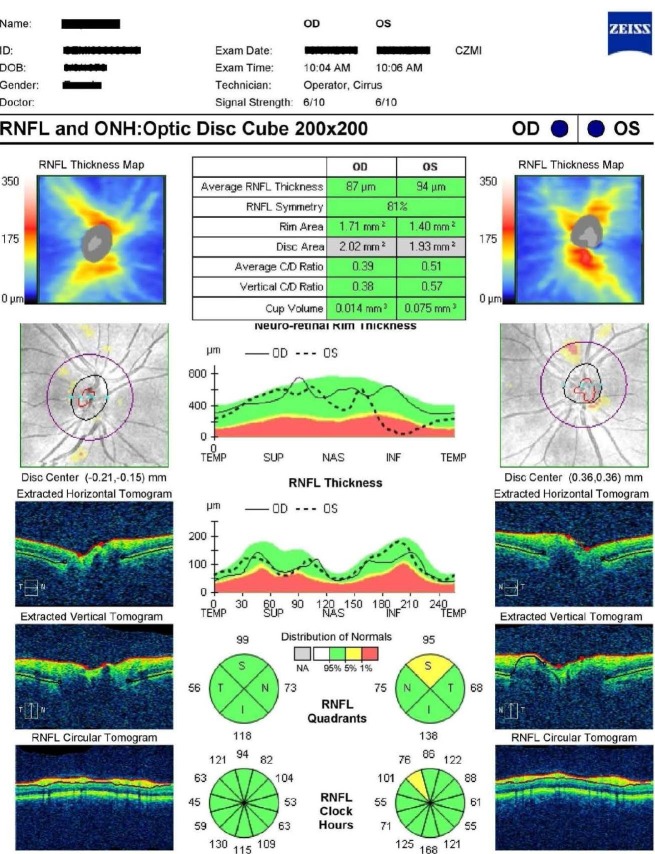
Optical Coherence Tomography. A patient with MS and no visual symptoms. Note the thinning of RNFL at the right eye (87 µm)

The thickness of the RNFL was shown to be reduced by 35,71% in the eyes of patients clinically unaffected of MS (**Fig. 1**).

The prevalence of VEP abnormalities in patients with clinically definite MS and no history of optic neuritis is higher than the incidence of peripapillary RNFL thinning.

**Fig. 2:**
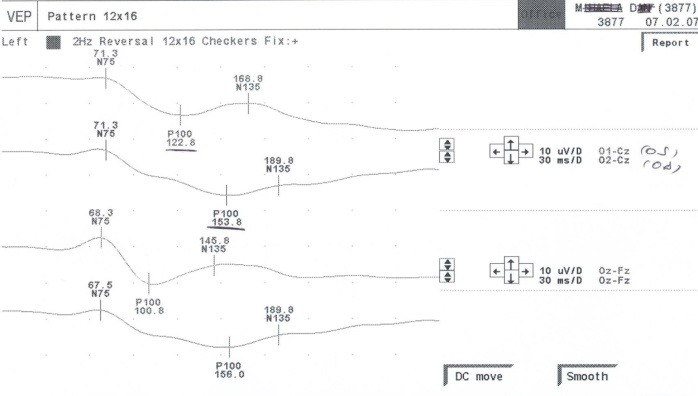
Visual evoked potential. A patient with MS with no subjective visual symptoms. Note the delayed latency of P100 wave over 115ms at both eyes

Of the fourteen patients, twelve (85,71%) were found with VEP delayed (P100 latencies >115 ms) (**Fig. 2**).

We found that VEP was more sensitive in detecting subtle subclinical defects than OCT (RNFL thickness).

## Discussion

Multiple sclerosis is a progressive disease in which subclinical RNFL thinning may occur, even in patients who have not been clinically diagnosed with optic neuritis [**17**-**19**].

The alteration of the visual evoked potential (VEP) latencies during pattern stimulation is considered one of the most characteristic electrophysiological signs in patients with MS, visually affected or unaffected [**20**]. Visual evoked potentials (VEP) remain the preferred test for the detection of clinical and subclinical optic neuritis [**1**].

The study showed that in patients affected by MS with no history of optic neuritis and no visual symptoms, there is a large prevalence of visual pathway involvement [**20**].

Clinically, it will be helpful to establish the baseline RNFL thickness and functional measurements in all MS patients at the time of the MS diagnosis. Change in RNFL thickness and/or visual function over time is likely the best approach in monitoring the disease progression [**2**]. The greatest predictor for RNFL in the unaffected eye was the RNFL in the fellow affected eye [**1**].

Based on OCT studies in MS, RNFL thickness is reduced significantly among patients (92 µm) versus controls (105 µm) and is particularly reduced in MS eyes with a history of optic neuritis (85 µm) [**3**].

Combining information from structural and functional tests and following individuals longitudinally is probably the best strategy for assessing and monitoring the optic nerve involvement in patients with MS [**3**].

The validation of OCT as an imaging biomarker in MS is important because several aspects of the information it generates are unique [**4**]. Imaging the RNFL allows a direct measurement of the unmyelinated axons of the central nervous system. The capacity to image central nervous system axons quickly and noninvasively, to minimize expense, and to correlate structural abnormalities with visual dysfunction add to the appeal of OCT as an imaging biomarker and outcome measure in clinical trials [**3**,**4**,**12**].

The important correlation between the functional and anatomic aspect confirms the value of VEP and OCT to appreciate the subclinical involvement of the optic nerve.
